# Does Diabetes Induce the Vascular Endothelial Growth Factor (VEGF) Expression in Periodontal Tissues? A Systematic Review

**DOI:** 10.3390/ijerph17082765

**Published:** 2020-04-16

**Authors:** Gianna Maria Nardi, Elisabetta Ferrara, Ilaria Converti, Francesca Cesarano, Salvatore Scacco, Roberta Grassi, Antonio Gnoni, Felice Roberto Grassi, Biagio Rapone

**Affiliations:** 1 Department of Dental and Maxillofacial Sciences, “Sapienza” University of Rome, 00161 Rome, Italy; giannamaria.nardi@uniroma1.it (G.M.N.); francesca.cesarano@gmail.com (F.C.); 2 Complex Operative Unit of Odontostomatology, Hospital S.S. Annunziata, 66100 Chieti, Italy; igieneeprevenzione@gmail.com; 3 Department of Emergency and Organ Transplantation, Division of Plastic and Reconstructive Surgery, “Aldo Moro” University of Bari, 70124 Bari, Italy; ilaria.converti@gmail.com; 4 Department of Basic Medical Sciences, Neurosciences and Sense Organs, “Aldo Moro” University of Bari, 70124 Bari, Italy; salvatore.scacco@uniba.it (S.S.); gnoniantonio@gmail.com (A.G.); feliceroberto.grassi@uniba.it (F.R.G.); 5 Department of Biomedical Sciences, University of Sassari, 07100 Sassari, Italy; grassi.roberta93@gmail.com

**Keywords:** vascular endothelial growth factor, periodontitis, type II diabetes, type I diabetes, periodontal disease

## Abstract

Aim: Diabetes and periodontal disease are both chronic pathological conditions linked by several underlying biological mechanisms, in which the inflammatory response plays a critical role, and their association has been largely recognized. Recently, attention has been given to diabetes as an important mediator of vascular endothelial growth factor (VEGF) overexpression in periodontal tissues, by virtue of its ability to affect microvasculature. This review aims to summarize the findings from studies that explored VEGF expression in diabetic patients with periodontitis, compared to periodontally healthy subjects. Materials and Methods: A systematic literature review was performed using the Preferred Reporting Items for Systematic Reviews and Meta-Analyses (PRISMA) guidelines. A PubMed search of select medical subject heading (MeSH) terms was carried out to identify all studies reporting findings about VEGF expression in periodontal tissues of diabetic patients up to May 2018. The inclusion criteria were studies on VEGF expression in periodontally diseased tissues of diabetic patients compared with nondiabetic subjects, with any method of analysis, and published in the English language. Results: Eight articles met the inclusion criteria. Immunohistochemistry was used in six of the studies, reverse transcriptase polymerase chain reaction (real-time RT-PCR) aiming to quantify mRNA VEGF expression was used in one study, and ELISA analysis was used for one study. Compared with nondiabetic patients, a higher VEGF expression in gingival tissue and gingival crevicular fluid (GCF) samples in diabetic patients with periodontitis was reported. Conclusions: Overall, novel evidence for the VEGF expression within the periodontal tissue of diabetic patients paves the way for further studies on the role of this protein in neovascularization physiology and pathophysiology in microvasculature of the periodontium.

## 1. Introduction

Diabetes and periodontal disease are both chronic pathological conditions linked by several underlying biological mechanisms, in which the inflammatory response plays a critical role, and their association has been largely recognized [1,2]. Periodontitis is an inflammatory disease affecting supporting tissues of teeth [3,4]. Its chronicization is a key determinant of clinical manifestations and underlying microvasculature alterations leading to irreversible damage [5]. It has been demonstrated that diabetic patients are more prone to developing periodontitis, and, conversely [6,7], uncontrolled diabetes is associated with higher risk for periodontitis onset and progression. Recently, in addition to the established relationship between periodontitis and diabetes, attention has been given to diabetes as an important mediator of vascular endothelial growth factor (VEGF) overexpression in periodontal tissues, by virtue of its ability to induce vasoreactivity in many organs [8,9,10,11]. Vascular endothelial growth factor (VEGF), a highly conserved heparin-binding homodimeric [12,13], disulfide-bound glycoprotein of 45kDa, is a key mediator of angiogenesis, a complex process which leads to the new growth of blood vessels from preexisting differentiated endothelial cells in the vascular network [14]. VEGF is a multifunctional growth factor implicated in embryonic development, and it is a powerful angiogenic agent in physiological and pathological neovascularization [13,14,15,16]. The vascular endothelial growth factor (VEGF) belongs to the endothelial growth factor family (VEGFs), currently consisting of seven known members with a regulatory role in different vascular systems: VEGF-A, VEGF-B, VEGF-C, VEGF-D, VEGF-E, VEGF-F, and PIGF [17]. VEGF exerts its biological effects through interaction with specific (VEGF-R) receptors, which belong to the receptor tyrosine kinase (RTK) subfamily [3,5]; the fms-like tyrosine kinase Flt-1 (VEGFR-1/Flt-1), the fetal liver kinase (VEGFR-2/KDR/Flk-1), and Flk-4 (VEGFR-3), causing proliferation of blood vessels (VEGF-A, VEGF-B, VEGF-C and VEGF-E) and lymphangiogenesis (VEGF-C and VEGF-D) [14,15,16]. The vascular endothelial growth factor receptors are one of the most important signaling pathways that regulate angiogenesis [17,18,19,20]. VEGFR-1 and VEGFR-2 are primarily implicated in this process, and there is a growing body of evidence suggesting that VEGFR-1 is involved in both physiological and pathological neovascularization [21,22,23,24,25]. The first evidence for the role of VEGFR-1 during angiogenesis came from the finding that abnormal blood vessel growth caused early embryogenic lethality when VEGFR-1 was targeted and deleted [2]. The negative role of VEGFR-1 in angiogenesis has been demonstrated by animal studies, observing an increase of endothelial cell progenitors and resulting in vascular disorganization as a consequence of loss of *VEGFR-1/flt-1* [26,27]. However, more recent in vitro evidence suggests that selective activation of *VEGFR-1 ligand is not* linked to the proliferation of endothelial cells [28]. While the precise role of VEGFR-1 in angiogenesis remains to be determined, the role of VEGFR-2 in neovascularization is well recognized [28,29,30]. Upon binding, VEGFR-2 homo- or heterodimerizes with monomer receptors, triggering autophosphorylation of its tyrosine residues with receptors that activate broad signaling cascades, leading to different biological responses involving the activation of receptor tyrosine kinase (RTKs) [30,31,32]. The binding of VEGF to its receptor promotes the activation of relay proteins that transmit a signal into the nucleus of the endothelial cell. Subsequently, the nuclear signal induces a group of genes to release molecules needed for new endothelial cell growth [33,34]. Among the biological actions of VEGF, a role for this molecule in the direct control function of periodontal damage in diabetic patients has been recently suggested [35,36,37]. Several lines of evidence confirm that VEGF is a positive regulator of angiogenesis in physiological and pathological conditions [35,38], stimulating extracellular matrix degradation, proliferation and migration of endothelial cells, and regulating vascular permeability [39,40,41,42,43]. Several factors have been demonstrated as inductors of mRNA VEGF transcription, including PDGF, EGF, TNF-α, TGF-β, and IL-1. Importantly, it has been found that VEGF levels are also regulated via the hypoxia exposure; the tissue tension oxygen induces the expression of VEGF irreversibly, through increased transcription and mRNA stabilization [44]. Pathological angiogenesis is correlated with diabetic microvasculopathy in many organs, playing a critical role in diabetic retinopathy [45,46], nephropathy [31,35], neuropathy [47], impaired collateral vessel formation, and other systemic conditions. Several factors related to diabetes lead to angiogenic stimulation, and, primarily, the vascular endothelial growth factor (VEGF) signaling pathway is involved [48,49]. Specifically, it has been demonstrated that diabetes causes defective VEGF signaling leading to impairment of tyrosine kinase receptors Flk-1 activation, the receptor implicated in different angiogenesis processes and in transmitting VEGF signaling [35]. This reduced activity results in increased serum VEGF levels, causing pathologic angiogenesis [15]. Sasso et al. [50] have shown that the reduction of Flt-1 and Flk-1 receptors influenced the VEGF expression in the myocardium of diabetic patients, leading to a greater expression when compared to healthy subjects. Waltenberger et al. [35] demonstrated that Flk-1 activation was abnormal in diabetic conditions. Furthermore, there other several factors involved in abnormal angiogenesis in diabetes, including: (a) a chronic inflammatory status with consequent secretions of pro-inflammatory molecules, characterizing diabetes mellitus, which increases VEGF transcription hypoxia-inducible factor-1α (HIF-1α) [51,52,53,54,55,56,57]; (b) the hypoxic condition, resulting in the upregulation of hypoxia inducible factors, which triggers cells to upregulate VEGF and other pro-angiogenic agents [58]; (c) the presence of oxidative stress, which has been shown to characterize diabetes, and is responsible for the secretion of proinflammatory cytokines such as TNF-α, transforming growth factors alpha (TGF-α) and beta (TGF-β), and interleukins 6 (IL-6) and 8 (IL-8); and (d) hyperglycemia and advanced glycation end products (AGEs), which contribute to impaired angiogenic potential [59,60,61,62,63,64] in vitro and other excess tissue factors.

## 2. Materials and Methods

A systematic literature review was performed using the Preferred Reporting Items for Systematic Reviews and Meta-Analyses (PRISMA) guidelines. A PubMed search of select medical subject heading (MeSH) terms to identify all studies that reported VEGF-expression in diabetic patients with periodontitis findings to May 2019 was performed: “vascular endothelial growth factors “ OR “vascular” AND “endothelial” AND “growth” AND “factors” OR “vascular endothelial growth factors” OR “vascular” AND “endothelial” AND “growth” AND “factors” OR “vascular endothelial growth factors” AND “diabetes mellitus” OR “diabetes” AND “mellitus” OR “diabetes mellitus” OR “diabetes” OR “diabetes insipidus” OR “diabetes” AND “insipidus” OR “diabetes insipidus” AND “periodontitis” OR “periodontitis”. To be eligible, every study had to include the assessment of the expression of vascular endothelial growth factor (VEGF) in diabetic patients with periodontal disease and provide a healthy comparison, including non-diabetic patients with or without periodontal disease. The following outcomes were extracted for each study, where available: type of analysis, number of patients, setting, and analysis method, as shown in Table 1 and Table 2.

## 3. Results

**Figure 1:**
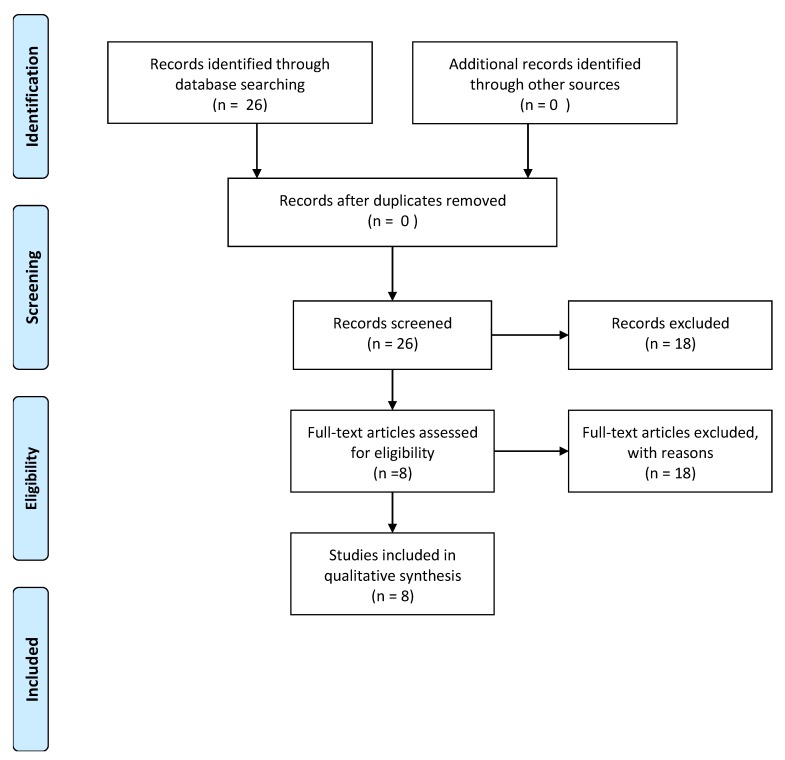
Flow diagram of information through the different phases of a systematic review.

The electronic search yielded a total of 26 articles. After examining the full text of articles, 18 of them were excluded from the review, as showed in Figure 1.

Of the eight remaining articles, six reported the results of immunochemistry quantification, one of ELISA analysis, and one real-time RT-PCR which aimed to quantify mRNA VEGF expression. A total of 330 patients were enrolled in the studies, and 424 gingival samples were analyzed. Furthermore, two studies also analyzed the VEGF expression into gingival crevicular fluid. The risk of bias for each study has been reported (Table 3).

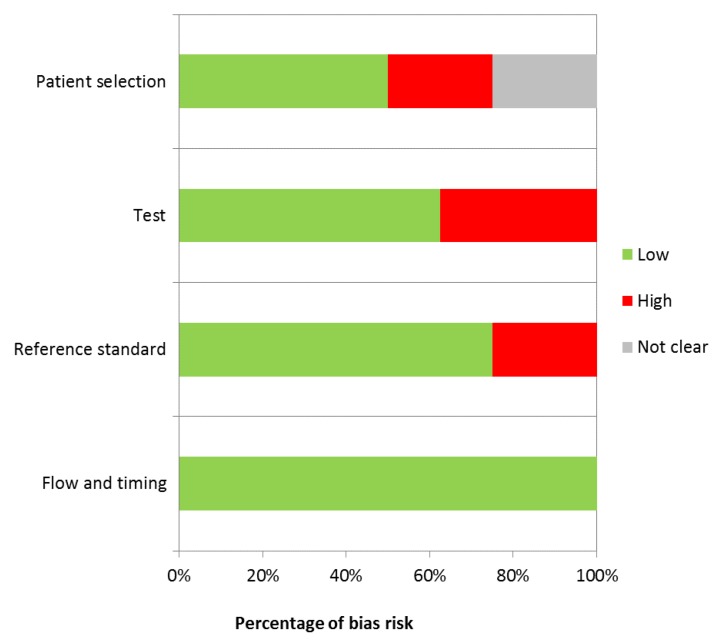



In the study conducted by Lucarini et al. [65], 16 gingival samples corresponding to non-diabetic patients with periodontitis and 32 gingival samples from diabetic patients with periodontitis were selected. As a control for specificity of detection, 16 gingival samples from healthy subjects without periodontitis were included in the study. The detection of expression of VEGF was conducted in epithelial cells and connective tissue cells and in endothelial cells of sub-epithelial connective tissue vessels by immunohistochemical labeling using specific monoclonal antibodies. Their analysis demonstrated that epithelial and endothelial cells of the diabetic patients’ group and periodontal group without diabetes showed strong VEGF immunostaining. Otherwise, endothelial cells and cells derived from the gingival epithelium of healthy subjects showed moderate immunostaining. The study of Aspriello et al. [66] described VEGF cytoplasmic expression by immunohistochemical staining (IHC) in endothelial cells of gingival tissue samples of 66 patients with periodontitis, including 22 patients with type II diabetes mellitus (T2DM), 22 patients with type I diabetes mellitus (T1DM), and 22 systemically healthy subjects. Cryostat sections of gingival tissue were immunostained with the monoclonal antibodies anti-VEGF. For the immunohistochemical study, the semiquantitative was used. The immunohistochemical data revealed a significant difference in the periodontal expression of VEGF among the study patients. There was significantly more immunoreactivity to VEGF in the epithelium of T1DM and T2DM than in the control group (*p* <0.05). Endothelial cells of patients with T1DM showed a greater mean (±SD) degree of immunostaining than those of patients with T2DM and patients without diabetes (*p* <0.05). Kranti et al. [67] conducted a similar analysis, assessing the expression of cytoplasmic VEGF in macrophages and fibroblasts. Fifty subjects were analyzed for VEGF expression by immunohistochemistry. Expression of both line cells VEGF was more moderate in healthy subjects with periodontitis and patients with controlled type II diabetes mellitus and periodontitis than in the periodontally healthy group (*p* <0.01). The discrepancies with the previous study are likely due to the fact that analysis of VEGF was taken after periodontal treatment, leading to a decrease in inflammatory response. In contrast, the one study that aimed to analyze the VEGF mRNA expression in gingival samples, using real-time RT-PCR, failed to detect this overexpression in diseased periodontal tissues of diabetic patients (Keles et al.) [68]. Specifically, a higher significantly positive amplification of the mRNA encoding VEGF was observed in healthy subjects than in periodontal groups with or without diabetes, while the amount of VEGF mRNA did not differ between the diseased groups, and no statistically significant difference was observed between the groups (*p* >0.05). This different response may be attributable to physiological angiogenesis in the periodontium vasculature. Ramya and colleagues [69] published the results of their research, showing that diseased periodontal sites in the diabetic patients were correlated with significantly higher levels of VEGF expression when compared to healthy sites, in accordance with the previous studies (*p* >0.001). The authors concluded that their findings may be due to insulin resistance, which appears to stimulate the induction of VEGF expression. On the other hand, Ünlü et al. [70] detected VEGF subcellular localization using immunochemistry methods. They reported that positive staining was significantly localized to cytoplasm of monocytes and macrophages in healthy tissue samples and periodontal sites of diabetic patients, compared to healthy subjects. Similarly, an increased VEGF expression in diabetic patients was observed by Sakallioglu et al. [71]. In their study, gingival tissues and GCF samples were drawn from analyzed sixteen type II diabetes mellitus patients under a good metabolic control condition (HbA1c value less than seven), and 15 patients under systemically healthy conditions, all with periodontitis. VEGF protein expression was analyzed by enzyme-linked immunosorbent assay (ELISA). Of interest, compared with the control group, a slightly higher VEGF concentration of GCF was observed in diabetic patients with periodontitis, and the difference was not statistically significant (*p* >0.05). The latter findings indicated an even stronger gingival tissue response to the diabetic condition when compared to the nondiabetic patients, showing a higher VEGF concentration, and showing that the intensity of this pro-angiogenic agent expression in periodontal tissues may be correlated with diabetes. These results were not in agreement with the previous study conducted by Güneri et al. [72] suggesting that VEGF expression in periodontal tissues is primarily dependent on the periodontal status and that diabetes might have an additive effect. The differences among the studies may be due to the characteristics of gingival tissue samples derived from different degrees of severity of periodontitis in the diabetic group, conditioning the VEGF expression. A comparison analysis between the studies was not possible because, although achieved staining by the immunohistochemistry technique was analogous to the results acquired with the ELISA test (enzyme-linked immunosorbent assay), the interpretation of immunohistochemistry expression is defined in a subjective manner, and the majority of authors have revealed the VEGF expression with subjective scoring, while the application of the ELISA test is established as an objective quantitative method.

## 4. Discussion

Angiogenesis is a multi-sequential process characterized by the interaction of a wide variety of regulatory mediators, in which vascular endothelial growth factor (VEGF) is a protein primarily involved. The processes underlying the pathogenesis of the microvascular and macrovascular complications of diabetes have been primarily ascribed to angiogenic factors. Vascular endothelial growth factors expression is induced by hyperglycemia, advanced glycation end products, and oxidative stress [72]. This response may result in pathologic neovascularization and altered vascular permeability, leading to detrimental effects in microvascular tissues, such as the retina. In addition, VEGF is involved in the pathogenesis of diabetic renal disease, resulting in increased expression in the epithelial cells of the glomerulus [73], podocyte [74], and collecting ducts. In addition, the clinical observations relating to diabetic cardiovascular disease have demonstrated a significant pathologic role of VEGF, showing a much greater expression in human arteriole smooth muscle cells and infiltrating macrophages after myocardial infarction (MI). Periodontal vasculature, during development of disease, is subjected to the microvasculature alterations which lead to supporting tissue destruction. There is increasing evidence that microvasculature of the periodontium is severely affected in diabetic patients with periodontitis, showing a higher vascular endothelial growth factor (VEGF) expression when compared to healthy subjects, with or without periodontitis. More recently, evidence has accumulated supporting the concept that microangiopathy induced by diabetes substantially contributes to periodontal vasculature alteration in periodontal tissues, inducing the VEGF expression through its capability to induce microvasculopathy in many organs. However, these results are in contrast with previous studies reporting that VEGF levels expression was increased in periodontal sites of systemically healthy patients, compared to patients without periodontitis [55]. Our review showed limited information. Limitations that may be encountered with this review include discrepancies associated with different VEGF expression quantification methods (Table 3). The interpretation of immunohistochemistry expression is generally made in a qualitative and subjective manner, whereas quantification is considered of little or no importance. A majority of studies used the qualitative (positive or negative) or semiquantitative (0, 1, 2, 3) interpretation of immunostaining, a key limitation of HIC staining. The analysis using staining scales is subject to interobserver variability and is often based on cellular presence or absence of a specific molecule. In addition, the evaluation of immunostaining may be influenced by heterogeneity of biologic VEGF expression between tissue samples and gingival crevicular fluid, which is susceptible to the saliva concentration fluctuation. In the majority of studies, a positive result referred essentially to the presence of brown staining (peroxidase), leading to confounding factors [41,42,43]. Similarly, two authors assessed VEGF expression analysis in which the results of the immunohistochemical investigation revealed marked cytoplasmic staining, but this analysis is considered to be non-specific. Additionally, difficulty in the interpretation and comparison of immunohistochemical studies included variability in patient selection, different immunohistochemical criteria used for determining the staining degree, and bias associated with the statistical method of analysis data. For instance, studies applying immunochemistry analysis revealed a globally increased VEGF expression in periodontal sites in diabetic patients compared to healthy subjects. Interestingly, although the reduced VEGF expression level in patients after periodontal treatment was obvious, this result might be of relevance, because improvement of inflammatory status has been shown to reduce the angiogenesis [45,54]. However, although from these results we can assume that the periodontal vasculature is affected by diabetes, and the VEGF expression may influence periodontal disease progression, further investigation establishing the degree of disorder at different disease stages of diabetic patients with periodontal disease are needed [63], because the duration and degree of hyperglycemia in diabetes may also be critical, as well as the periodontal status. Nonetheless, as suggested by this, we cannot exclude the involvement of preexisting periodontal inflammation, as the author hypothesized that preexisting periodontal disease in patients with diabetes might partly explain increased VEGF expression and impact on outcome.

## 5. Conclusions

The current paradigm of linkage between type 2 diabetes and periodontitis must be integrated. We recognize an additional factor impacting the risk of periodontitis in patients with diabetes that may inform this discussion of new paradigms. This review has highlighted the detrimental effect of diabetes on periodontal tissues, resulting also from the induction of overexpression of VEGF, responsible for pathological angiogenesis. To date, an extremely limited number of studies have addressed the overexpression of VEGF in the periodontium of diabetic patients, and partially conflicting results have been reported. Expression of VEGF in diabetic patients with periodontitis has been demonstrated [21], but no conclusive data on inductive effect of periodontal damage have been published. However, the pivotal role of diabetes, and its signaling system in periodontal tissues, remains largely unexplored. Although expression of VEGF in the periodontium has been preliminarily reported, further investigations are needed for an appropriate conclusion in this linkage. In addition, clinical trials exploring new treatment strategies that target both diseases are required.

## Figures and Tables

**Table 1:** Data extraction table.

STUDY	Fusun Unlu (2003)	Pelin Guneri (2004)	Elif Eser Sakallioglu (2007)	Simone Domenico Aspriello (2009)
**PURPOSE**	Determine the association between VEGF expression in healthy and periodontally diseased tissues of healthy and diabetic patients	Compare VEGF expression in healthy and periodontally diseased tissues with gingival crevice fluid (GCF) of healthy persons and diabetic patients	Demonstrate that DM may have an inductive effect on the VEGF levels of periodontium during periodontal disease	Investigate VEGF expression and microvessel density (MVD) in patients with periodontitis with and without DM
**SETTING**	Department of Oral Diagnosis and Radiology Department of Ege University School of Dentistry, TURKEY	Department of Oral Diagnosis and Radiology Department of Ege University School of Dentistry, TURKEY	Department of Periodontology, Faculty of Dentistry, Kurupelit, TURKEY	Division of Periodontology, Department of Clinical and Dental Sciences, Polytechnic University of Marche, Torrette, Ancona, ITALY
**SUBJECTS**	n = 20 12 male, 8 females Mean age = 52.6 years	n = 20 12 male, 8 females Mean age = 52.6 years	n = 31 male Mean age = 50.58 ± 1.54 years	n = 66 Age range, 47 to 71 years
**INCLUSION CRITERIA**	Previously diagnosed of type 2 DM, no smokers, patients with a diagnosis of periodontitis	Previously diagnosed of type 2 DM, no smokers, patients with a diagnosis of periodontitis	Previously diagnosed of type 2 DM with a disease history of 2–5 years, no smokers, HbA1c’s values less than 7, patients with a diagnosis of periodontitis	Diagnosis of generalized, severe, chronic periodontitis, no smokers, ≥20 teeth and bone loss >50% in all teeth, absence of important systemic diseases, absence of any ongoing drug therapy, relatively stable glycemic control, HbA1c levels in the non-diabetic range (<6.1%) in the assay used for this study, fasting plasma glucose <6.0 mmol/l
**EXCLUSION** **CRITERIA**	Patients with a DM history of less than 5 years, with any other systemic diseases, or who were under additional medication	Patients with a DM history of less than 5 years, with any other systemic diseases, or who were under additional medication	Females, patients with any other systemic diseases, insulin replacement	None of the participants had taken antibiotics, corticosteroids, or non-steroidal anti-inflammatory drugs within the 6 months prior to treatment or undergone periodontal treatment during the previous 2 years, and no individuals included in this study suffered from hemoglobinopathies capable of altering the values of HbA1c
**DESIGN**	Observational Case-Control Study	Observational Case-Control Study	Observational Case-Control Study	Observational Case-Control Study
**INDIPENDENT VARIABLES**	HgA1c, PD, AL	HgA1c, PI, PD, AL	PI, GI, PD, GR, AL	PI, GI, PD, AL
**DEPENDENT VARIABLES**	VEGF levels	VEGF levels	VEGF levels	VEGF levels
**ANALYSIS METHOD**	Immunohistochemical analyses	Immunohistochemical analyses	Enzyme-linked immunosorbent assay (ELISA), Biochemical analysis	Immunohistochemical analysis
**STATISTICAL SIGNIFICANCE**	In the negative control group tissue samples, no VEGF expression was observed. Among the 10 systemically healthy people, no evidence of VEGF was observed in healthy gingival samples but was found in diseased tissues in 2 cases. In the diabetic patients, VEGF was observed in 4 healthy gingival tissues and in 6 periodontal sites. (*p* = 0.043)	No VEGF staining was observed in the negative control group or in the systemically healthy people’s healthy tissue samples, whereas four samples of diabetic patients showed positive staining (*p* < 0.05). However, VEGF was revealed in two tissue samples of periodontal sites of systemically healthy people and in six samples of the diabetic patients (*p* > 0.05). In all test groups, GCF VEGF levels were higher in periodontal sites (*p* < 0.05) than in healthy sites.	The VEGF concentrations of gingival supernatants in the test group were higher than in the control group. The mean gingival VEGF level was 55.89 ± 8.11 pg/mL in the test and was 24.81 ± 2.04 pg/mL in the control group, and the difference between the groups was statistically significant (*p* < 0.01). The mean VEGF level of GCF in the test group was 38.96 ± 4.89 pg/mL, and the mean VEGF level of GCF in the control group was 32.20 ± 4.02 pg/mL. The difference in the VEGF levels of GCF was not statistically significant between the groups (*p* > 0.05)	The three groups showed no significant differences in the prevalence of gender and sites of gingival biopsies. Moreover, no significant differences in clinical and periodontal parameters were found among the groups. Immunohistochemical VEGF expression was observed in all gingival samples at the cellular cytoplasmic level. In controls, immunohistochemical analysis showed diffuse VEGF-positive epithelial cells (39.32–12.72) mainly in the basal layer. In the dermis, a modest percentage of VEGF-positive cells (19.86–2.23) was found; in particular, VEGF-positive endothelial cells were numerous (38.82–6.73). VEGF-positive cells were significantly increased in the epithelium of patients with T1DM (68.55–22.54) and T2DM (55.36–19.99) compared to controls (*p* < 0.05), whereas no significant differences were found among the three groups for VEGF expression in dermal cells. A significant increase of VEGF-positive endothelial cells (49.18–10.17) was observed in patients with T1DM compared to patients with T2DM and controls (*p* < 0.05). CD34 immunostaining was observed in all gingival samples. The mean percentage of MVD was 27.40–11.28 in patients with T1DM and was statistically increased compared to controls (19.57–3.55; *p* < 0.05) and patients with T2DM (12.42–6.39; *p* < 0.001).

**Table 2:** Data extraction table.

STUDY	Gonca Cayir Keles (2010)	Ramya, Senthil Kumar (2014)	K. Kranti (2015)	Guendalina Lucarini (2016)
**PURPOSE**	Determine vascular endothelial growth factor (VEGF) mRNA expression levels in gingival tissues of gingivitis and periodontitis patients with diabetes mellitus and those without	Determine the association between VEGF expression in healthy and periodontally diseased tissues of healthy and diabetic patients	Evaluate immunohistochemically vascular endothelial growth factor (VEGF) and Ki-67 in human gingival samples and to compare these factors between healthy and diabetic patients	Investigate the relation between vascular endothelial growth factor (VEGF) and inducible nitric oxide synthase (iNOS) in periodontal disease of diabetic and nondiabetic individuals
**SETTING**	Department of Periodontology, Faculty of Dentistry, Ondokuzmayis University, Samsun, TURKEY	Division of Periodontics, Rajah Muthiah Dental College & Hospital, Annamalai University	Department of Periodontics, M S Ramaiah Dental College and Hospital, Bangalore, Karnataka	Dental Clinic of the Department of Clinical Special and Dental Science (Polytechnic University of Marche, Ancona, Italy)
**SUBJECTS**	Forty-five subjects, 30–66 years of age	n = 34 40 to 56 years	n = 50 18 to 65 years	n = 64 47 to 71 years
**INCLUSION CRITERIA**	Criteria for gingivitis were (1) gingival index (GI) C2, (2) probing pocket depth (PPD) 4 mm with bleeding on probing, (3) clinical findings such as red color, swelling of the gingival margin without bone resorption or periodontal pocketing, (4) no proximal sites with clinical attachment loss, (5) no signs of attachment and bone loss observed by clinical and radiographic examination. Criteria for periodontitis were (1) GI C2, (2) PPD C4 mm with bleeding on probing, (3) clinical and radiographic attachment and bone loss. Criteria for type II diabetes were (1) a history of the disease of at least 5 years, (2) mean hemoglobin A1c value of\7, (3) no insulin therapy, (4) absence of any other systemic diseases. Healthy controls without any systemic diseases showed clinically healthy gingiva without bleeding (depth of gingival sulcus \3 mm) and no evidence of periodontal pocketing, clinical attachment loss or bone resorption	Patients all diagnosed as chronic periodontitis, based on the probing pocket depth, clinical attachment loss (CAL), plaque index, type II diabetic and nondiabetic patients with age group between 40 to 56 years, with mean HbA1c value ranging between 6 to 8 and duration not less than five years	Periodontally healthy patients with no sites with pocket depth and clinical attachment levels >3 mm and <20% of sites with gingival bleeding and bleeding on probing; patients with periodontally diseased gingiva without any systemic disease and patients with periodontally diseased gingiva with controlled type II DM	Inclusion criteria were (1) age ≥18 years, (2) absence of smoking habit, (3) absence of major diseases, (4) no history of allergies, and (5) no use of antibiotics, corticosteroids, or non-steroidal anti-inflammatory drugs within the 6 months prior to inclusion in the study or periodontal treatment during the previous 2 years
**EXCLUSION CRITERIA**	Systemic diseases (i.e., hypertension, hyperlipidemia or coronary heart disease), chronic high dose steroid therapy, radiation or immune suppressive therapy, chronic usage of anti-inflammatory drugs, pregnancy, lactation, smoking or periodontal treatment in the previous 6 months	Smokers, history of any systemic diseases and medications influencing the study	Subjects who were pregnant and lactating, on antibiotic therapy, smokers, on long term administration of anti-inflammatory medication, on localized radiation therapy of the oral cavity and on antineoplastic chemotherapy	No individuals included in this study suffered from hemoglobinopathies capable of altering the HbA1c values
**STUDY DESIGN**	Observational Case-Control Study	Randomized clinical trial	Observational Case-Control Study	Observational Case-Control Study
**INDIPENDENT VARIABLES**	GI, PD, HbA1c	PI, GI PD, CAL	PI, GI, PD, CAL, HbA1c	HbA1c, PI, GI, PD, CAL
**DEPENDENT VARIABLES**	VEGF mRNA levels	VEGF levels	VEGF levels, Ki-67 levels	VEGF levels, i NOS levels
**ANALYSIS METHOD**	Real-time RT-PCR	Immunohistochemical Analysis	Immunohistochemical Analysis	Immunohistochemical Analysis
**STATISTICAL SIGNIFICANCE**	There was no significant difference in age and gender distribution between the groups (*p* > 0.05). The PPD, CAL, GI, and PI values were not statistically different between groups 1 and 3 nor between groups 2 and 4 (*p* > 0.05). The findings of GI and PI were not statistically different between groups 1, 2, 3, and 4. Expression of VEGF mRNA was detected in all study groups. There was no significant difference in the expression levels of VEGF mRNA between the groups (*p* > 0.05)	This study showed the expression of VEGF was found to be higher in the diseased sites of diabetic patients with chronic periodontitis when compared to their healthy sites. which was found to be statistically significant with *p*-value > 0.001 as assessed by the degree of staining of monocytes and endothelial cells immunohistochemically. There was a high degree of positive staining of monocytes with an increase in the mean number of cells (50–60%) when compared to endothelial cells (22–33%) and was found to be statistically significant with *p*-value > 0.001	The present study found moderate intensity staining for VEGF in periodontitis group and periodontitis with controlled type II DM group and mild intensity staining for VEGF in periodontally healthy group. With regard to Ki-67, negative staining was observed in periodontally healthy group and mild staining in periodontitis group and periodontitis with controlled type II DM group	No significant difference among the four groups was found in age. As expected, glycemia and HbA1c were significantly higher in diabetic patients than in non-diabetic ones. No difference between DM and DM+CP group was found in these two parameters. PI was statistically significantly higher in DM+CP group than in the other groups, whereas GI was higher in both periodontitis groups than in the other two. PD, CAL, and bone loss were similar in CP and DM+CP groups, and, as expected, higher than non-periodontitis groups

**Table 3:** Evaluation of bias risk.

Evaluation of Bias Risk				
	Patient Selection	Test	Reference Standard	Flow and Timing
**Fusun Unlu (2003)**	Not clear	High	High	Low
**Pelin Guneri (2004)**	Low	Low	Low	Low
**Elif Eser Sakallioglu (2007)**	High	Low	Low	Low
**Simone Domenico Aspriello (2009)**	Not clear	High	Low	Low
**Gonca Cayir Keles (2010)**	Low	Low	Low	Low
**Ramya, Senthil Kumar (2014)**	High	High	Low	Low
**K. Kranti (2015)**	Low	High	High	Low
**Guendalina Lucarini (2016)**	Low	High	Low	Low
Low	4	5	6	8
High	2	3	2	0
Not clear	2	0	0	0
